# Hospitals under 50 Beds

**Published:** 1918-12-21

**Authors:** 


**Hospital Needs and Hospital Appeals—(*continued*).**

### Hospitals under 50 Beds.

**THE BELGRAVE HOSPITAL FOR CHILDREN, CLAPHAM ROAD, S.W.**—This hospital's work has been carried on undiminished, and—thanks to generous friends—its income has been well maintained; but, owing to the high prices of food, drugs, and in fact all necessities, there is already a deficiency of £1,900 this year. Mr. Alfred J. Small is the Acting Secretary.

**THE METROPOLITAN EAR, NOSE, AND THROAT HOSPITAL, FITZROY SQUARE, W.**—This hospital, now the oldest of its specialty, is pressingly in need of funds. During the past year it has treated numerous pensioner patients, a department which promises considerable extension. A large sum is owing to its bankers. The Secretary is Mr. John Mackinna.

**PADDINGTON GREEN CHILDREN'S HOSPITAL, W. 2.**—This hospital with its forty-six cots is tucked away in the north-east corner of Paddington Green. It has also an invaluable convalescent home at Slough, accommodating sixteen patients in winter and twenty-four in summer. The in-patients average over 700 a year, and the out-patients' attendances reach nearly 50,000. The committee earnestly appeal for subscriptions, donations, and legacies

**Hospital Needs and Hospital Appeals**—(*continued*).

to enable them to "save the babies." Visitors will be shown round the hospital any afternoon from two to four. Contributions will be thankfully received by the Chairman, Sir Douglas Owen, K.B.E., or the Secretary, Mr. F. Stanley Cheer, at the hospital.

**ROYAL WESTMINSTER OPHTHALMIC HOSPITAL.**—This hospital is in very great need of funds at the present time, the demands made upon its resources having been greatly increased owing to so many of the eye departments at the general hospitals having been closed. Average number of beds occupied: Civilian cases twenty-five, soldiers ten, total thirty-five. Mr. Conrad Thies is the Acting Secretary.

**ST. JOHN'S HOSPITAL FOR DISEASES OF THE SKIN, 49 LEICESTER SQUARE, W.C. 2.**—It will be most difficult to continue the work of the hospital unless immediate help be forthcoming. Many discharged soldiers who have faithfully served their King and country are now under treatment at this hospital. The Secretary is Mr. George A. Arnaudin.

**ST. PAUL'S HOSPITAL, RED LION SQUARE, HOLBORN, W.C. 1.**—Saint Paul's was the first Hospital founded in the United Kingdom for the treatment of the allied skin and genito-urinary (including venereal) diseases. The Hospital is always open in the evening, as well as in the afternoon, so that patients can attend without interference with their work and wages. Rebuilding is contemplated and an appeal for £10,000 for this purpose and for £1,600 a year for maintenance is made.

**ST. PETER'S HOSPITAL, COVENT GARDEN.**—With a record of splendid achievement behind it, and a wide field of fruitful effort open before it, the hospital is unfortunately in such financial straits that its work is in danger of being crippled, if not stopped altogether. Contributions, however small, will therefore be thankfully acknowledged. Secretary, Mr. Irwin H. Beattie.

**THE MEMORIAL HOSPITAL, MILDMAY PARK, N. 1,** is situated in a quiet corner, surrounded by the compound of the old Conference Hall, and supplies a much-felt need in this portion of London. Patients come for treatment from all parts of the country, and appreciate the peace and restfulness to be found here. The hospital contains thirty beds. All communications should be sent to the Secretary, Memorial Hospital, Mildmay Park, N. 1.

